# N-Substituted indole carbohydrazide derivatives: synthesis and evaluation of their antiplatelet aggregation activity

**DOI:** 10.1186/s40199-014-0065-6

**Published:** 2014-09-20

**Authors:** Seyedeh Sara Mirfazli, Farzad Kobarfard, Loghman Firoozpour, Ali Asadipour, Marjan Esfahanizadeh, Kimia Tabib, Abbas Shafiee, Alireza Foroumadi

**Affiliations:** Department of Medicinal Chemistry, Faculty of Pharmacy and Pharmaceutical Sciences Research Center, Tehran University of Medical Sciences, Tehran, Iran; Department of Medicinal Chemistry, School of Pharmacy, Shahid Beheshti University of Medical Sciences, Tehran, Iran; Phytochemistry Research Center, Shahid Beheshti University of Medical Sciences, Tehran, Iran; Drug Design and Development Research Center, Tehran University of Medical Sciences, Tehran, Iran; Neuroscience Research Center, Institute of Neuropharmacology, Kerman University of Medical Sciences, Kerman, Iran

**Keywords:** Anti-platelet aggregation, Indole, *N*-acylhydrazone

## Abstract

**Background:**

Platelet aggregation is one of the most important factors in the development of thrombotic disorders which plays a central role in thrombosis (clot formation). Prophylaxis and treatment of arterial thrombosis are achieved using anti-platelet drugs. In this study, a series of novel substituted indole carbohydrazide was synthesized and evaluated for anti-platelet aggregation activity induced by adenosine diphosphate (ADP), arachidonic acid (AA) and collagen.

**Methods:**

Our synthetic route started from methyl 1H-indole-3-carboxylate (1) and ethyl 1H-indole-2-carboxylate (4) which were reacted with hydrazine monohydrate 99%. The aldol condensation of the later compound with aromatic aldehydes led to the formation of the title compounds. Sixteen indole acylhydrazone derivatives, **3d-m** and **6d-i** were tested for anti-platelet aggregation activity induced by adenosine diphosphate (ADP), arachidonic acid (AA) and collagen.

**Results:**

Among the synthesized compounds, **6g** and **6h** with 100% inhibition, proved to be the most potent derivatives of the 2-substituted indole on platelet aggregation induced by AA and collagen, respectively. In 3-substituted indole **3m** with 100% inhibition and **3f** and **3i** caused 97% inhibition on platelet aggregation induced by collagen and AA, respectively.

**Conclusion:**

In this study, compounds **6g, 6h, 3m, 3f** and **3i** showed better inhibition on platelet aggregation induced by AA and collagen among the title compounds. Quantitative structure–activity relationship (QSAR) analysis between the structural parameters of the investigated derivatives and their antiplatelet aggregation activity was performed with various molecular descriptors but, analysis of the physicochemical parameters doesn’t show a significant correlation between the observed activities and general molecular parameters of the synthesized derivatives. Although, due to the existence of several receptors on the platelets surface which are responsible for controlling the platelet aggregation, the investigated compounds in the present study may exert their activities through binding to more than one of these receptors and therefore no straight forward SAR could be obtained for them.

## Background

Cardiovascular diseases are responsible for the largest number of death and disability worldwide. Platelet adhesion and aggregation are key events in hemostasis and thrombosis which cause disrupted atherosclerotic plaques that is the initiator of most thrombotic disorders including heart attacks and strokes [1-3]. Platelets play the major role in the pathogenesis of thromboembolic disorders and activation of the platelets by complex biochemical pathways and mediators is the primary step in this process [4,5]. Endogenous agonists such as arachidonic acid (AA), adenosine 5′-diphosphate (ADP) that acts on purinergic receptors on the platelet-known as P_2_Y receptors, thromboxane A_2_ (TxA_2_), thrombin, platelet activating factor (PAF), epinephrine (EPN) and collagen are among potent agonists that initiate the formation of stable platelet aggregates [6-8].

Clinical evidence has clearly proven that antiplatelet aggregation agents are useful for preventing thrombotic disorders. On the other hand, there are still some serious limitations to currently use agents which include weak inhibition of platelet function (aspirin), slow onset of action (clopidogrel), variable response to treatment among patients and high incidence of bleeding events which is dose dependent in both aspirin and clopidogrel drug therapy [9]. Considering the current situation, pursuit of finding novel scaffolds as new antiplatelet aggregation drugs which are more effective and safer with fewer side effects is very important [10].

A novel group of heterocyclic acylhydrazone derivatives with antiplatelet aggregation activity on rabbit platelet-rich plasma have been reported [11,12]. Furthermore, the *N*-acylhydrazone (NAH) moiety, have shown a series of biological activities such as analgesic, anti-inflammatory [13-20], protozoa proteases inhibition [21], HIV-1 reverse transcriptase dimmer destabilization [22], antibiotic and antifungal activities [23], and cardiovascular actions [24-28].

**Figure 1:**
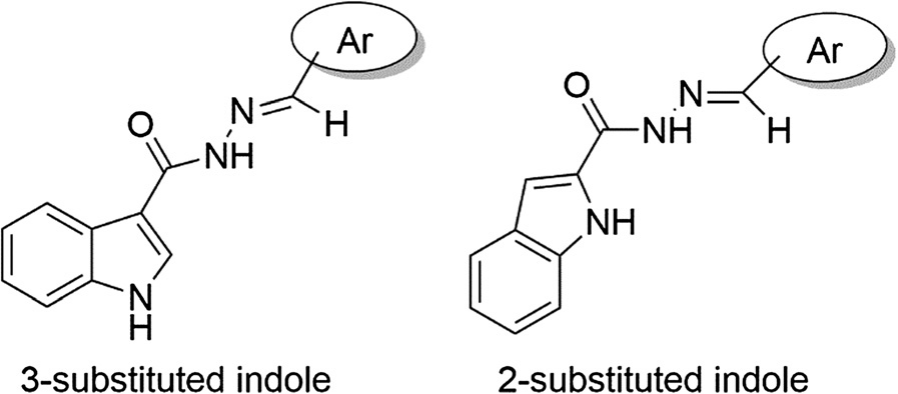
**Schematic representation of the general hydrazone structural backbone with antiplatelet activity.**

Indole ring is another structural moiety which has been reported to have antiplatelet aggregation activity [29]. Considering this background, a diverse group of derivatives have been synthesized in this study by molecular hybridization between indole and hydrazone moieties, to find the structure–antiplatelet activity relationship of the derivatives. The schematic structural backbone for these compounds which contain both indole and *N*-acylhydrazone is depicted in Figure 1.

### Chemistry

**Scheme 1:**
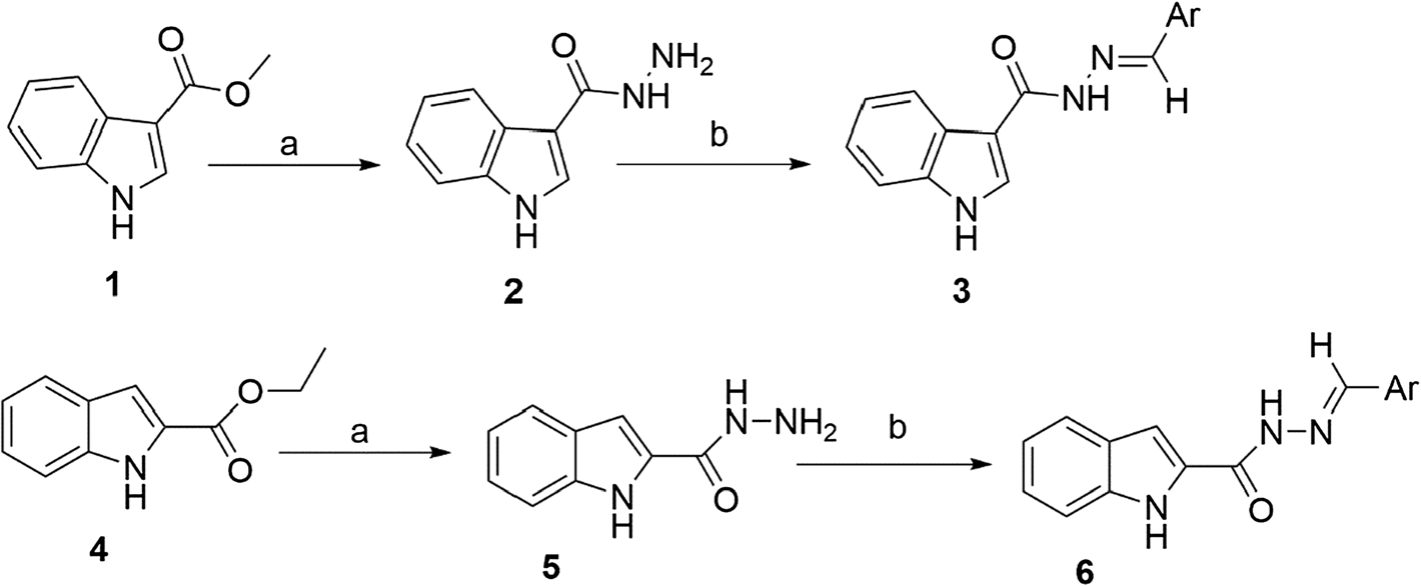
**The synthesis pathway for indole** ***N*** **-acylhydrazones.** Reagents and reaction condition: **a)** Hydrazine monohydrate 99% (NH_2_NH_2_), Ethanol (a few drop), reflux at 80°C, 3 h **b)** ArCHO, H_2_O, Glacial acetic acid (a few drop), reflux at 100°C, 3 h.

The synthetic procedure planned to obtain the desired indole *N*-acylhydrazone derivatives, is shown in Scheme 1. The key intermediates were obtained by hydrazinolysis of **1** and **4** in 96% and 91% yield, respectively, using hydrazine monohydrate 99% in ethanol. The final indole *N*-acylhydrazone derivatives were obtained by condensing the hydrazide intermediates with the proper aromatic aldehydes (ArCHO) in water and glacial acetic acid as the solvent, in good yields.

## Material and methods

### General

All commercial solvents, chemicals and reagents were purchased from either Merck or Sigma-Aldrich with the highest purity and used without further purification. Proton nuclear magnetic resonance (^1^H NMR) spectra were recorded on a Bruker 500 MHz spectrometers (Bruker, Rheinstetten, Germany) and pick positions are illustrated in parts per million (*δ*) in DMSO-*d*_*6*_ solution and tetramethylsilane (0.05% v/v) as internal standard and coupling constant values (*J*) are given in Hertz. Signal multiplicities are reported by: s (singlet), d (doublet), t (triplet), q (quadruplet), m (multiplet) and br (broad signal). For NMR spectral data assignments, the atom numbering of compounds is depicted in Table 1. Analytical thin layer chromatography (TLC) was performed with Merck silica gel plates and visualized with UV irradiation (254 nm) or iodine. Electrospray ionization mass spectra (ESI-MS) were obtained using Agilent 6410 Triple Quad. LC/MS. Melting points were obtained by an Electrothermal 9100 apparatus and are uncorrected. The IR spectra were taken by a Perkin-Elmer 843 spectrometer with KBr as diluent. The elemental analysis for C, H and N was performed by a Costech model 4010 and the percentage values agreed with the proposed structures within ±0.4% of the theoretical values. All described products showed ^1^H NMR spectra according to the assigned structures. The physicochemical parameters including Clog *P* value, surface area, molecular volume, refractivity and polarizability were calculated by Hyperchem 8.0 software.

**Table 1:** **Effect of 3-substituted indole (3d-m) and 2-substituted indole (6d-i) derivatives at 1 mM concentration on** ***in-vitro*** **platelet aggregation induced by AA, ADP and collagen**

**Derivative**	**Structure**	**AA**	**ADP**	**Collagen**
		**Inhibition (%)** ^**b**^		
**3d**		30.1 ± 3	31 ± 1.5	24.5 ± 2.1
**3e**		94 ± 5	20 ± 1.3	80 ± 4.3
**3f**		97 ± 4.9	41.5 ± 2.1	73.6 ± 2.9
**3 g**		94 ± 2.5	46 ± 2.5	65.6 ± 3.1
**3h**		95 ± 3.9	21.3 ± 1	44.6 ± 2.1
**3i**		97 ± 5.1	47.5 ± 1.2	74 ± 3.9
**3j**		96 ± 4.6	42.8 ± 0.9	81 ± 1.9
**3 k**		93 ± 1.9	55 ± 1.3	91 ± 2.7
**3 l**		94 ± 3	53 ± 2.1	91 ± 0.9
**3 m**		94 ± 2.8	35 ± 1.2	100 ± 2.0
**6d**		96 ± 4.1	31.4 ± 1.4	61.6 ± 3.2
**6e**		35 ± 2.5	66.8 ± 1.1	8 ± 0.9
**6f**		94.5 ± 3.1	25.9 ± 0.6	61 ± 3
**6 g**		100 ± 3.8	26.8 ± 1	27.3 ± 1.2
**6 h**		96 ± 2.9	24 ± 0.8	100 ± 3.4
**6i**		98 ± 1.4	51 ± 1.3	80 ± 2.5
**Indomethacin** ^**a**^		100 ± 4.3	42 ± 1.1	100 ± 2.8
**Aspirin** ^**a**^		100 ± 2.8	21 ± 0.6	100 ± 3.1

^a^Aspirin and Indomethacin were used as a positive control.

^b^Values are presented as mean ± S.E. of three separate determination.

#### General procedure for the preparation of carbohydrazides (2, 5)

Compounds (**1** or **4**) (2.86 mmol) was added to a solution of hydrazine monohydrate 99% (2.14 mL; 2.18 g; 43.6 mmol) in ethanol (0.5 mL) and the reaction mixture was stirred at about 80°C temperature, for 2 h. TLC indicated the end of reaction. The mixture was cooled by addition of a water/ice mixture. The solid was filtered in excellent yield (Scheme 1) [30-32].

1*H*- indole-3-carbohydrazide (**2**) and 1*H*- indole-2-carbohydrazide (**5**) were prepared according to a literature method [30-32].

#### General procedure for the preparation of N-acylhydrazone derivatives

Equimolar amount of appropriate aromatic aldehyde was added to a solution of hydrazide compound (**2** or **5**) in 10 mL of water, in presence of catalytic amount of glacial acetic acid (0.4 mL). Reaction mixture was heated under reflux with stirring for about 2 h and poured into ice/water mixture. The precipitate was filtered and washed with cold water (Scheme 1).

*N*’-(4-hydroxybenzylidene)-1*H*-indole-3-carbohydrazide (**3i**), *N*’-(3-hydroxybenzylidene)-1*H*-indole-3-carbohydrazide (**3j**), *N*’-benzylidene-1*H*-indole-3-carbohydrazide (**3 m**), *N*’-(2-hydroxybenzylidene)-1*H*-indole-2-carbohydrazide (**6e**), *N*’-(2-methoxybenzylidene)-1*H*-indole-2-carbohydrazide (**6f**) and *N*’-benzylidene-1*H*-indole-2-carbohydrazide (**6i**) were prepared according to a literature method [30-32].

#### N’-(2-hydroxybenzylidene)-1H-indole-3-carbohydrazide (3d)

Yield: 92%, mp 256- 259°C. IR (KBr) cm^-1^: 3365 (ν OH), 3283, 3041, 2927, 1660, 1614, 1596, 1577, 1564. ^1^H NMR (500 MHz, DMSO): *δ* 11.79 (s, 1H, CONH), 11.71 (bs, 1H, Indole NH), 11.51 (bs, 1H, OH), 8.52 (s, 1H, ─N═C*H*─) 8.21 (bs, 1H, ─N═CH─C_6_*H*_5_, H_2_), 8.20 (d, 1H, *J* = 7.85 Hz, ─N═CH─C_6_*H*_5_ ,H_4_), 7.52 (d, 1H, *J* = 7.5 Hz, ─N═CH─C_6_*H*_5_, H_6_), 7.50 (d, 1H, *J* = 7.8 Hz, ─N═CH─C_6_*H*_5_, H_4_), 7.29 (td, 1H, *J* = 7.0, 1.40 Hz, Indole H_7_), 7.21 (td, 1H, *J =* 7.4, 1.45 Hz, Indole H_5_), 7.17 (td, 1H, *J =* 7.0, 1.45 Hz, Indole H_6_), 6.95- 6.91 (m, 2H, ─N═CH─C_6_*H*_5_, H_3,_ H_5_), ESI-Mass *m/z*: 280 [M + H]^+^, 302 [M + Na]^+^; Anal. Calcd. for C_16_H_13_N_3_O_2_: C, 68.81; H, 4.69; N, 15.05. Found: C, 68.64; H, 4.83; N, 14.92.

#### N’-(2-nitrobenzylidene)-1H-indole-3-carbohydrazide (3e)

Yield: 96%, mp 272- 274°C. IR (KBr) cm^-1^: 3282, 3218, 3143, 3089, 1635, 1595, 1564, 1540 and 1353 (NO_2_). ^1^H NMR (500 MHz, DMSO): *δ* 11.83 (s, 1H, CONH), 11.81 (s, 1H, Indole NH), 8.72 (s, 1H, ─N═CH─C_6_*H*_5_, H_2_), 8.28 (bs, 1H, ─N═C*H*─), 8.21 (d, 1H, J = 7.8 Hz, Indole H_4_), 8.16 (d, 1H, J = 7.4 Hz, ─N═CH─C_6_*H*_5,_ H_6_), 8.08 (dd, 1H, *J* = 7.20, 1.0 Hz, ─N═CH─C_6_*H*_5_, H_3_), 7.83 (t, 1H, *J =* 7.5 Hz, ─N═CH─C_6_*H*_5_, H_5_), 7.66 (td, 1H, *J* = 7.6, 1.35 Hz, ─N═CH─C_6_*H*_5_, H_4_), 7.49 (d, 1H, *J* = 7.9 Hz, Indole H_7_), 7.23-7.16 (m, 2H, Indole H_5_, H_6_), ESI-Mass *m/z*: 309 [M + H]^+^, 331 [M + Na]^+^, 347 [M + K]^+^; Anal. Calcd. for C_16_H_12_N_4_O_3_: C, 62.33; H, 3.92; N, 18.17. Found: C, 62.58; H, 4.08; N, 18.32.

#### N’-(2-methoxybenzylidene)-1H-indole-3-carbohydrazide (3f)

Yield: 69%, mp 229- 231°C. IR (KBr) cm^-1^: 3300- 3200 (ν NH), 3112, 3076, 1622, 1601, 1578, 1540; ^1^H NMR (500 MHz, DMSO): *δ* 11.73 (s, 1H, CONH), 11.41 (s, 1H, Indole-NH), 8.65 (bs, 1H, Indole H_2_ ), 8.22 (bs, 2H, Indole H_4_, ─N═C*H*─), 7.87 (d, 1H, *J* = 6.70 Hz, ─N═CH─C_6_*H*_5_, H_6_), 7.48 (d, 1H, *J* = 7. 85 Hz, Indole H_7_), 7.41 (td, 1H, *J =* 7.3, 1.35 Hz, ─N═CH─C_6_*H*_5_, H_4_), 7.20 (t, 1H, *J =* 7.0 Hz, ─N═CH─C_6_*H*_5_, H_5_), 7.15 (t, 1H, *J =* 7.4 Hz, Indole H_5_), 7.12 (d, 1H, *J =* 8.3 Hz, ─N═CH─C_6_*H*_5_, H_3_), 7.04 (t, 1H, *J* = 7.4 Hz, Indole H_6_), 3.89 (s, 3H, ─OCH_3_); ESI-Mass *m/z*: 294 [M + H]^+^, 316 [M + Na]^+^; Anal. Calcd. for C_17_H_15_N_3_O_2_: C, 69.61; H, 5.15; N, 14.33. Found: C, 69.46; H, 5.33; N, 14.12.

#### N’-(3-chlorobenzylidene)-1H-indole-3-carbohydrazide (3 g)

Yield: 78%, mp 288- 291°C. IR (KBr) cm^-1^: 3545, 3390, 3320, 3263, 3068, 1635, 1580, 1558,1548; ^1^H NMR (500 MHz, DMSO): *δ* 11.78 (s, 1H, CONH), 11.50 (s, 1H, Indole-NH), 8.27 (bs, 1H, ─N═C*H*─), 8.21 (s, 1H, Indole H_2_), 8.20 (s, 1H, Indole H_4_), 7.78 (s, 1H, ─N═CH─C_6_*H*_5_, H_2_), 7.68 (d, 1H, *J* = 7.0 Hz, ─N═CH─C_6_*H*_5_, H_6_), 7.51- 7.47 (m, 3H, Indole H_7_, ─N═CH─C_6_*H*_5_, H_4_, H_5_), 7.22- 7.15 (m, 2H, Indole H_5_, H_6_); ESI-Mass *m/z*: 298 [M + H]^+^, 320 [M + Na]^+^; Anal. Calcd. for C_16_H_12_ClN_3_O: C, 64.54; H, 4.06; N, 11.91. Found: C, 64.19; H, 4.24; N, 11.76.

#### N’-(4-chlorobenzylidene)-1H-indole-3-carbohydrazide (3 h)

Yield: 74%, mp 265- 267°C. IR (KBr) cm^-1^: 3394, 3240, 3060, 1637, 1603, 1555, 1536; ^1^H NMR (500 MHz, DMSO): *δ* 11.76 (s, 1H, CONH), 11.45 (s, 1H, Indole-NH), 8.35- 8.21 (m, 3H, ─N═C*H*─, Indole H_2_, H_4_), 7.75 (d, 2H, *J =* 8.5 Hz, ─N═CH─C_6_*H*_5_, H_2_, H_6_), 7.53 (d, 2H, *J* = 8.5 Hz, ─N═CH─C_6_*H*_5_, H_3_, H_5_), 7.49 (d, 1H, *J =* 8.0 Hz, Indole H_7_), 7.22- 7.15 (m, 2H, Indole H_5_, H_6_); ESI-Mass *m/z*: 298 [M + H]^+^, 320 [M + Na]^+^; Anal. Calcd. for C_16_H_12_ClN_3_O: C, 64.54; H, 4.06; N, 11.91. Found: C, 64.43; H, 3.91; N, 12.16.

#### N’-(2-fluorobenzylidene)-1H-indole-3-carbohydrazide (3 k)

Yield: 90%, mp 239- 240°C. IR (KBr) cm^-1^: 3299- 3073 (ν NH), 3032, 2956, 1636, 1614, 1586, 1555. ^1^H NMR (500 MHz, DMSO): *δ* 11.77 (s, 1H, CONH), 11.50 (bs, 1H, Indole NH), 8.55 (bs, 1H, ─N═C*H*─), 8.22 (d, 1H, *J =* 7.5 Hz, Indole H_4_), 7.94 (t, 1H, *J* = 6.8 Hz, ─N═CH─C_6_*H*_5_, H_4_), 7.50- 7.45 (m, 3H, ─N═CH─C_6_*H*_5_, H_6_, Indole H_2_, H_7_), 7.33- 7.29 (m, 2H, ─N═CH─C_6_*H*_5_, H_3_, H_5_), 7.21 (td, 1H, *J* = 6.5, 1.3 Hz, Indole H_5_), 7.16 (td, 1H, *J =* 6.5, 1.3 Hz, Indole H_6_); ESI-Mass *m/z*: 282 [M + H]^+^, 304 [M + Na]^+^; Anal. Calcd. for C_16_H_12_FN_3_O: C, 68.32; H, 4.30; N, 14.94. Found: C, 68.64; H, 4.13; N, 14.62.

#### N’-(3-fluorobenzylidene)-1H-indole-3-carbohydrazide (3 l)

Yield: 87%, mp 278- 281°C. IR (KBr) cm^-1^: 3319- 3200 (ν NH), 3139, 3089, 1647, 1591, 1558, 1500. ^1^H NMR (500 MHz, DMSO): *δ* 11.76 (s, 1H, CONH), 11.50 (bs, 1H, Indole NH), 8.34- 8.27 (m, 3H, ─N═C*H*─, Indole H_4_, H_2_), 7.57- 7.48 (m, 4H, ─N═CH─C_6_*H*_5_ ,H_2_, H_5_, H6, Indole, H_7_), 7.28- 7.24 (m, 1H, ─N═CH─C_6_*H*_5_, H_4_), 7.21 (td, 1H, *J* = 6.8, 1.2 Hz, Indole H_5_), 7.16 (td, 1H, *J =* 6.8, 1.2 Hz, Indole H_6_); ESI-Mass *m/z*: 282 [M + H]^+^, 304 [M + Na]^+^; Anal. Calcd. for C_16_H_12_FN_3_O: C, 68.32; H, 4.30; N, 14.94. Found: C, 68.14; H, 4.03; N, 15.02.

#### N’-(2-fluorobenzylidene)-1H-indole-2-carbohydrazide (6d)

Yield: 98%, mp 186- 188°C. IR (KBr) cm^-1^: 3450, 3227, 3038, 2922, 1643, 1621, 1612, 1593, and 1564. ^1^H NMR (500 MHz, DMSO): *δ* 12.03 (s, 1H, CONH), 11.85 (s, 1H, Indole NH), 8.71 (s, 1H, ─N═C*H*─), 7.98 (t, 1H, *J =* 7.3 Hz, ─N═CH─C_6_*H*_5_ ,H_4_), 7.70 (d, 1H, *J* = 7.8 Hz, ─N═CH─C_6_*H*_5_,H_6_), 7.52- 7.47 (m, 2H, Indole H_4_, H_7_), 7.35- 7.32 (m, 2H, Indole H_5_, H_6_), 7.24 (t, 1H, *J* = 7.4 Hz, ─N═CH─C_6_*H*_5_, H_3_), 7.08 (t, 1H, *J =* 7.4 Hz, ─N═CH─C_6_*H*_5_, H_5_); ESI-Mass *m/z*: 282 [M + H]^+^; Anal. Calcd. for C_16_H_12_FN_3_O: C, 68.32; H, 4.30; N, 14.94. Found: C, 68.14; H, 4.63; N, 15.12.

#### N’-(3-fluorobenzylidene)-1H-indole-2-carbohydrazide (6 g)

Yield: 88%, mp 171- 173°C. IR (KBr) cm^-1^: 3448, 3313, 3264, 3126, 3071, 1629, 1597, 1577, 1529; ^1^H NMR (500 MHz, DMSO): *δ* 12.02 (s, 1H, CONH), 11.84 (s, 1H, Indole NH), 8.47 (s, 1H, ─N═C*H*─), 7.70 (d, 1H, *J =* 8.0 Hz, ─N═CH─C_6_*H*_5_ ,H_2_),7.61 (d, 1H, *J =* 7.3 Hz, Indole H_7_), 7.58- 7.52 (m, 2H, ─N═CH─C_6_*H*_5_ H_5_, Indole, H_4_), 7.48 (d, 1H, *J =* 8.0 Hz, ─N═CH─C_6_*H*_5_, H_6_), 7.34 (s, 1H, Indole H_3_), 7.30 (t, 1H, *J =* 8.5 Hz, ─N═CH─C_6_*H*_5_, H_6_), 7.24 (t, 1H, *J =* 7.5 Hz, Indole H_6_), 7.08 (t, 1H, *J =* 7.5 Hz, Indole H_5_); ESI-Mass *m/z*: 282 [M + H]^+^, 304 [M + Na]^+^; Anal. Calcd. for C_16_H_12_FN_3_O: C, 68.32; H, 4.30; N, 14.94. Found: C, 68.01; H, 4.33; N, 14.62.

#### N’-(3-hydroxybenzylidene)-1H-indole-2-carbohydrazide (6 h)

Yield: 89%, mp 278- 281°C. IR (KBr) cm^-1^: 3412 (ν OH), 3227, 3185, 3048, 2924, 1624, 1599, 1583, 1564, 1507; ^1^H NMR (500 MHz, DMSO): *δ* 12.05 (s, 1H, CONH), 11.84 (bs, 2H, Indole NH, OH), 8.44 (s, 1H, ─N═C*H*─), 7.82 (s, 1H, ─N═CH─C_6_*H*_5_, H_2_), 7.74- 7.68 (m, 2H, Indole H_4_, H_7_), 7.53- 7.51 (m, 2H, ─N═CH─C_6_*H*_5_, H_5_, H_6_), 7.47 (d, 1H, *J =* 8.3 Hz, ─N═CH─C_6_*H*_5_, H_4_), 7.34 (s, 1H, Indole H_3_), 7.24 (t, 1H, *J =* 7.1 Hz, Indole H_6_), 7.08 (t, 1H, *J =* 7.1 Hz, Indole H_5_); ESI-Mass *m/z*: 280 [M + H]^+^, 302 [M + Na]^+^; Anal. Calcd. for C_16_H_13_N_3_O_2_: C, 68.81; H, 4.69; N, 15.05. Found: C, 69.04; H, 4.41; N, 14.92.

### Biological assay

#### In vitro evaluation of anti-platelet aggregation activity

Human plasma used to measure the derivatives anti-platelet aggregation activity. Fresh blood was obtained from healthy volunteer with negative history of drug consumption from 15 days prior to the test. Platelet-rich plasma (PRP) was obtained from citrated whole blood (9:1 by volume) which centrifuged at 1,000 rpm for 8 min. The remained layer was centrifuged at 3,000 rpm for 15 min and the upper layer; PPP (Platelet poor plasma) was collected as the blank. The platelet count was adjusted to 250,000 plts/mL by diluting PRP with appropriate amount of PPP. To the PRP samples, test compounds previously dissolved in DMSO (at 0.05% final concentration) were added and samples were incubated for 5 min at 37°C. Then ADP (5 μM), collagen (1.25 mg/mL) or AA (1.25 mg/mL) was added and platelet shape change and aggregation were monitored for 5 min. DMSO (0.5% v/v) was used as negative control and aspirin and indomethacin were applied as standard drugs. The extent of platelet aggregation was calculated by the following formula:

$$ \mathrm{Inhibition}\% = \left[1\hbox{-} \left(\mathrm{D}/\mathrm{S}\right)\right]\ast 100 $$

D = platelet aggregation in the presence of test compoundsS = platelet aggregation in the presence of solvent.

The platelet aggregation inhibitory activity was expressed as percent inhibition by comparison with that measured for the vehicle (DMSO) alone and IC_50_ values were obtained from log (concentration) − inhibition (%) diagram and was defined as the concentration of the test compound that inhibits the platelet aggregation by 50%. Data were presented as mean ± S.E.M. of three independent experiments performed in triplicate. IC_50_values and inhibition data were analyzed with prism software.

### Consent

The study was approved in the Institute Review Board with code number 93-6-10:1–1. Written informed consent was obtained from the patient for the publication of this report and any accompanying images.

## Results

The synthetic pathway is disclosed in Scheme 1. Final desired derivatives were prepared by a two-step procedure. The structures were confirmed by spectroscopic techniques including IR, Mass and ^1^H NMR. Molecular mass of all the derivatives was determined by Electron-spray ionization mass spectrometry (ESI–MS) as M + 1 and/or M + 23 relating to hydrogen and sodium adducts of the intact molecules, respectively. All the synthesized compounds were evaluated for their ability to inhibit platelet aggregation of human platelet-rich plasma (PRP) induced by AA, ADP and collagen as potent aggregation inducers, and using indomethacin and aspirin were applied as standard drugs. The results of *in-vitro* antiplatelet aggregation activity for the title compounds were summarized in Table 1. All the derivatives were initially tested at 1 mM.

The physicochemical parameters of the derivatives were calculated and are listed in Table 2.

**Table 2:** **General molecular parameters of the synthesized compounds**

**Compound**	**Clog P**	**R** ^**a**^	**P** ^**b**^	**V** ^**c**^	**SA** ^**d**^	
					**Approx.**	**Grid**
**3d**	2.92	86.69	30.1	771.23	374.67	469.41
**3e**	-0.15	92.78	32.03	809.38	404.94	487.59
**3f**	3.35	88.16	30.83	781.07	373.02	472.72
**3g**	2.72	90.56	31.99	854.73	356.93	517.77
**3h**	3.85	91.28	32.12	807.06	402.79	488.39
**3i**	3.28	86.69	30.1	772.67	378.22	470.61
**3j**	2.72	88.16	30.83	784.59	381.64	474.45
**3k**	2.72	88.16	30.83	783.92	379.99	474.45
**3l**	3.85	91.28	32.12	807.9	403.8	488.87
**3m**	3.14	86.56	30.19	764.21	367.69	464.55
**6d**	3.26	87.96	30.1	775.75	381.25	476.6
**6e**	3.69	89.44	30.83	787.22	379.72	479.81
**6f**	3.06	94.21	32.66	844.8	422.2	515.34
**6g**	3.62	87.96	30.1	778.08	384.8	477.59
**6h**	3.06	89.44	30.83	791.05	387	482.43
**6i**	3.48	87.83	30.19	772.17	374	474.2

^a^Refractivity.

^b^Polarizability.

^c^Molecular volume.

^d^Surface area.

## Discussion

### Chemistry

All derivatives of 3-substituted indole and 2-substituted indole were obtained by the reaction of **2** and **5** with the proper aldehydes. *Synthesis of Schiff bases were* performed in ethanol with a few drops of glacial acetic acid. This reaction in the majority of the cases was straight forward; however, the products were soluble in ethanol and their separation was difficult. Therefore in another effort, the solvent was changed to water, a few drops of glacial acetic acid was added to the reaction mixture and heated for 10 min. After completion of reaction, the products were obtained in excellent yields.

In the ^1^H NMR spectra of these compounds the existence of two singlet at 11.00 to 12.00 ppm was assigned to hydrazide NH and indole NH. Also, singlet signal at 8.20-8.80 ppm was assigned to H─C = N. The ^1^H NMR and ESI-mass data of compounds approved the exact structures.

### Antiplatelet aggregation activity

Platelet activation and thrombus formation are major causes of cardiovascular diseases and thrombosis. Thus, antiplatelet therapy is a useful way to prevent or treat these diseases; these diseases; thus, antiplatelet agents such as aspirin, ticlopidine and dipyridamole have been clinically used for thrombus-related diseases [9]. However, the side effects of mentioned agents frequently have been reported and a new group of compounds with greater efficacy and safety are desired. Therefore, in the present study, the inhibitory effects of synthesized compounds on platelet aggregation were evaluated by turbidimetric method reported by Born and Cross [33] using APACT 4004 aggregometer. The baseline value was set using PRP and maximal transmission using PPP. Compounds **3d-m** and **6d-i** were tested for anti-platelet aggregation activity induced by adenosine diphosphate (ADP), arachidonic acid (AA) and collagen using indomethacin and aspirin as standards.

Interestingly, most of the tested derivatives selectively inhibited platelet aggregation induced by AA and collagen with satisfactory percent inhibition values. According to the literature [15]; herein, antiplatelet aggregation activity of *N*-acylhydrazones is probably related to modulation of AA cascade enzymes.

Among the synthesized indole-2-carbaldehyde derivatives compound **6g** exhibited 100% inhibition of platelet aggregation at 1 mM when AA was used as agonist while this compound has no significant inhibitory activity against ADP and collagen induced platelet aggregation. Comparing the results obtained for indole derivatives, compounds **3m** and **6h** showed the best antiplatelet aggregation effect which induced by collagen. On the other hand, effects of the synthesized compounds on the platelet aggregation induced by ADP shows another pattern: all the compounds caused no significant inhibition on platelet aggregation except **6e** which showed 66.7% inhibition.

The IC_50_ values were calculated for more potent compounds (**3f, 3i, 3k, 3l, 3m, 6d, 6g, 6h** and **6i**) for the inhibition of AA and collagen-induced aggregation which are shown in Table 3.

**Table 3:** **IC**_**50**_ **values for the antiplatelet aggregation activity induced by collagen and AA** ^**a**^

**Compound**	**Aryl**	**IC**_**50**_ **(μM)** ^**b**^	
		**AA**	**Collagen**
**3f**	2-methoxyphenyl	310±8.1	121.5±3.1
**3i**	4-hydroxyphenyl	290±5.3	188±2.8
**3k**	2-fluorophenyl	179±2.4	122±1.6
**3l**	3-fluorophenyl	321±3.9	120±4.0
**3m**	phenyl	182±5.2	21±0.9
**6d**	2-fluorophenyl	286±1.5	720±9.1
**6g**	3-fuorophenyl	140±4.3	>1000
**6h**	3-hydroxyphenyl	200±2.0	190±3.2
**6i**	phenyl	94±1.9	134±4.1
Indomethacin		3±0.2	1.2±0.1
Aspirin		30.3±2.6	9.7±0.6

^a^Data related to compounds 3 and 6 as shown in Scheme 1.

^b^IC_50_ values represent mean ± S.E. of triplicate measurements from one of three independent experiments.

However, the obtained results were compared with those reported by Kobarfard et al. on antiplatelet aggregation effect of some indole derivatives [4]. It was found that the insertion of acyl group to indole hydrazone moiety cannot improve platelet aggregation inhibitory activity.

In order to investigate the possible relationship between the structural parameters of the investigated derivatives and their antiplatelet aggregation activity, quantitative structure–activity relationship (QSAR) analysis was performed with various molecular descriptors. The calculated octanol–water partition coefficient (Clog P) has been considered as descriptor for the hydrophobic effect. The steric effect has been described by means of the surface area (SA: approx and grid) and molecular volume (V) refractivity (R) and polarizability (P) have been used as descriptors for both volume and electronic state (London dispersive forces) properties of the molecules. For each descriptor, the best multilinear regression equation was obtained. The calculated physicochemical parameters of the derivatives are listed in Table 2. Analysis of the physicochemical parameters doesn’t show a significant correlation between the observed activities and general molecular parameters of the synthesized derivatives.

## Conclusion

In summary, we have synthesized sixteen *N*-acylhydrazone derivatives (**3d-m** and **6d-i**) and evaluated their antiplatelet aggregation activity against collagen, ADP and AA as the aggregation inducers. Compounds **3e**, **3g**, **3h**, **3i**, **3j**, **3k**, **3l**, **3m**, **6d**, **6f**, **6g, 6h** and **6i** showed significant antiplatelet aggregation (>90%) when arachidonic acid was used as the inducer. While, **3l, 3k, 3m** and **6h** exhibited best (>90%) platelet aggregation inhibition induced by collagen among other compounds.

Failure to extract a clear correlation between activities and general molecular parameters of the synthesized compounds could be related to the existence of several receptors on the platelets surface which are responsible for controlling platelet aggregation. Platelets are activated by variety of metabolic pathways. The mechanism of platelet aggregation pathway is very complex and involves multiple components and it can be controlled by heterogeneous group of endogenous compounds such as ADP, ATP, collagen, tryptophan, epinephrine, thromboxane A_2_ and calcium. Each can independently and together begin the process leading to platelet aggregation. These compounds on platelets have specific receptors and the investigated compounds in the present study may exert their activities through binding to more than one of these receptors and therefore no straight forward SAR could be obtained. The findings of this study will be helpful for the development of new antiplatelet compounds providing some directions in the area of antiplatelet drug discovery.
